# Schizotypal Traits, Psychopathology, and Reflective Functioning Impairments During Adolescence: A Bayesian Network Approach

**DOI:** 10.1093/schbul/sbae041

**Published:** 2025-03-04

**Authors:** Eduardo Fonseca-Pedrero, Alicia Pérez-Albéniz, Beatriz Lucas-Molina, Susana Al-Halabí, Martin Debbané

**Affiliations:** Department of Educational Sciences, University of La Rioja, Logroño, Spain; Department of Educational Sciences, University of La Rioja, Logroño, Spain; Department of Developmental and Educational Psychology, University of Valencia, Valencia, Spain; Department of Psychology, University of Oviedo, Oviedo, Spain; Developmental Clinical Psychology Research Unit, Faculty of Psychology and Educational Sciences, University of Geneva, Geneva, Switzerland; Research Department of Clinical, Educational and Health Psychology, University College London, London, UK

**Keywords:** schizotypy, reflective functioning, mentalization, network analysis, directed acyclic graphs, adolescence

## Abstract

**Background:**

New theoretical and measurement models related to Bayesian networks can usefully be implemented to enrich our understanding of psychosis risk. The present study aims to explore, using a directed acyclic graph (DAG), the putative causal relationship within schizotypal facets, as well as between schizotypal dimensions, psychopathology, and reflective functioning (RF) impairments, in a representative sample of non-clinical adolescents.

**Study Design:**

A sample of 1476 adolescents from the general population participated in a cross-sectional survey. The Oviedo Schizotypy Assessment Questionnaire-Revised, the Strengths and Difficulties Questionnaire, and the Reflective Functioning Questionnaire (RFQ) were used.

**Study Results:**

Schizotypal traits were positively associated with psychopathology and hypomentalizing. Putative causal relationships are presented between Reality distortion, Social disorganization, and Anhedonia. In addition, estimated DAG suggests that schizotypal dimensions influence psychopathology and RF impairments.

**Conclusions:**

The findings suggest different pathways connecting schizotypal traits, mental health problems, and RF impairments during adolescence. The use of probabilistic DAG may allow us to make more robust conclusions about the direction of causation and to unravel potentially complex causal chains in the study of psychosis risk.

## Introduction

Increased attention is currently directed to understanding the nature of risk for schizophrenia spectrum and other psychotic disorders (SSPDs).^1,2^ The incorporation of new preventive, contextual, staging, and transdiagnostic approaches may help to improve the understanding of SSPDs.^3^ New studies need to adopt dynamic, complex systems, and mechanism-based perspectives rather than static, common latent cause, and disorder-based approaches. Incorporating these approaches into the field of schizotypy may contribute to our knowledge of tentative etiological mechanisms of psychosis in order to develop preventive strategies.^4^

Schizotypy is defined as a latent personality organization reflecting a putative liability for schizophrenia spectrum disorders.^5^ Schizotypy can manifest itself, at the phenotypic level, with a variety range of expressions, such as schizotypal traits, psychotic-like experiences, subclinical psychotic symptoms, psychotic symptoms, and psychosis-spectrum disorders.^6,7^ Beyond representing a risk for schizophrenia spectrum disorders, schizotypy is a genuine manifestation of its latent liability.^6^ Previous studies have found that schizotypal personality is a multidimensional phenotype, most often composed of three factors, like those found in individuals with psychosis. The three-factor model characterized by Positive (eg, hallucinations, ideas of reference, magical thinking, paranoid ideation), Interpersonal (eg, blunted affect, social anxiety, lack of close friends), and Disorganized (eg, odd behavior and speech) dimensions has been replicated across samples and tools.^8^

Previous studies have demonstrated that schizotypal traits at the population level may represent the behavioral manifestation of distributed multifactorial risk for psychosis.^9–12^ For instance, subtle, non-psychotic forms of perceptual aberrations or anhedonia are at an increased risk for SSPDs outcomes.^13,14^ In this regard, Radua et al^15^ conducted an umbrella review and found that the subclinical expression of the psychosis phenotype as an ultra-high risk state (UHR) for psychosis and trait anhedonia were among the main risk factors for psychosis with convincing or highly suggestive evidence of association. The predictive power of clinical interviews used in UHR samples is very comparable to that found in preventive medicine^16^; however, previous studies have found that childhood psychotic symptoms were not specific to a diagnosis of schizophrenia in adulthood.^17^ The mere presence of schizotypal traits or psychotic-like experiences is neither necessary nor a sufficient condition for the later development of SSPDs outcomes, other mental problems (eg, depression), or poorer functioning.^9,11^

Novel theoretical and measurement models have to be tested and refined in order to capture the complexity of unfolding psychosis phenotype. A network theory of mental disorders has been proposed.^18,19^ From this framework, mental disorders constitute emergent properties that arise from causal relations among symptoms (eg, behaviors, mental states, signs, traits). The network approach provides an alternative way to conceptualize psychosis and related phenomena (ie, schizotypal personality) as complex dynamic systems.^20^ This interactional approach focuses on specific symptoms and signs and their interrelationships rather than latent diseases. According to network theory, dynamic interactions between symptoms of mental diseases play an important role in their formation and maintenance. Although network theory has frequently been contrasted with the paradigm of assuming common causes underlying symptoms of mental diseases and employing latent variable models, it has recently been proposed that the distinction between network and common cause models may not be as apparent as previously thought. Thus, the proposed latent network model has been provided to combine the latent variable model and the network model.^21^ Emerging network analysis techniques provide new potential ways of modeling and understanding psychopathological processes and mental disorders,^22,23^ in particular, psychosis phenotype.^24–26^

Several psychometric analyses are available to estimate networks.^27,28^ However, in cross-sectional designs, estimated networks describe the predictive power and relative importance of distinct variables, but not causality. In this context, directed acyclic graph (DAG) models are tools for describing causal relationships and for guiding attempts to learn them from data. Bayesian networks are probabilistic graphical models widely employed to understand dependencies in high-dimensional data, and even to facilitate causal discovery.^29^ Causal graphs are based on strong assumptions (eg, causal sufficiency).^30^ As a result, several studies have used DAGs in psychosis^31–34^ and psychopathology research.^35,36^ According to Moffa et al^32^ probabilistic DAGs represent all the variables and links in a full picture of a unique network model, locating variables in a putative causal cascade in which upstream variables are potential causes of downstream variables.^33^ DAGs can also provide better insight into potential causal and complex relationships between multiple variables (within and across domain levels) and better understand the complexity of the mechanisms that connect the variables. The logic behind using a DAG to depict hypothetical causal structures is explained elsewhere.^37,38^

Prior research has shown that emotional and behavioral symptoms are a key factor in all stages of psychotic phenomena (eg, prodrome, UHR, first episode).^12,39,40^ It is already well documented that, along the extended psychosis phenotype, schizotypal traits, psychotic-like experiences, and subclinical psychotic symptoms have cross-sectional and longitudinal relationships with anxiety and depression symptoms.^41–44^ For instance, individuals who meet the UHR criteria often have concurrent mood and/or anxiety disorders.^45^ At the population level, those individuals who report schizotypal traits or psychotic-like experiences and who also refer to internalizing or externalizing psychopathology may indicate an underlying vulnerability to SSPDs or may be at risk for later psychosis.^46,47^

Reflective functioning (RF) may also play a relevant role in psychosis risk.^48,49^ RF, or mentalizing, refers to the ability to understand others and the self in terms of internal mental states.^50,51^ Mentalizing processes are more generally part of social cognitive processing. Mentalizing is thought to play a key role in preventing the development of mental health disorders such as psychosis, or attenuating their functional impact.^48,52^ For example, good performance on the RF was found to be a predictor of attenuation of early risk symptoms such as delusional ideation in children with aberrant perceptual experiences.^53^ On the other hand, low RF, characterized by an inability to understand behaviors in terms of mental states, has been associated with schizotypy and mental health problems.^54^ Both emotional dysregulation and RF impairment may then serve as significant candidate targets for psychological treatments or prevention strategies for psychosis.^49^

To date, despite the large body of research, the functional relationship between schizotypal traits, psychopathology, and RF impairments, during adolescence through the lens of the Bayesian network approach has not been analyzed. Thus, the mechanisms by which schizotypal traits are related to emotional dysregulation or RF impairments need to be further explored. In addition, further research is warranted to develop a better understanding of the facets of schizotypal and their associations with potential risk factors such as hypomentalizing or psychopathology, beyond the traditional categorical approach that has led to a simplified and incomplete view of psychological problems such as psychosis.

Within this research framework, the main goal of the present study was to explore, using a Bayesian network approach, the putative causal relationship within schizotypal facets, as well as between the schizotypal dimensions, psychopathology, and RF impairments, in a representative sample of non-clinical adolescents. The use of probabilistic DAG may allow for more robust conclusions about the direction of causation and provide a new perspective on this complex comorbidity. In addition, the Bayesian network model allows us to unravel potentially complex causal chains. Our intentions in this article were to demonstrate the potential of DAG in improving our understanding of schizotypy, as well as to characterize the functional relationships among schizotypal traits and psychopathology in the development of preventive interventions for individuals at risk for psychosis.

## Methods

### Participants

The total student population of La Rioja (a region in northern Spain) was sampled using stratified random sampling at class level. The students came from different public and non-public educational institutions, compulsory secondary education, and vocational training. Strata were developed based on the type of educational institution (public or non-public), as well as on the educational level. A total of 1476 students, 663 men (44.9%), and 813 (55.1%) women, from 34 schools and 98 classrooms participated in the study. The mean age was 16.13 years (SD = 1.36), ranging from age 14 to 19 years (14 years, *n* = 200; 15 years, *n* = 312; 16 years, *n* = 366; 17 years, *n* = 363, 18 years, *n* = 167; 19 years, *n* = 68). The participants belonged to different nationalities: 89.9% were Spanish, 3.7% were Latin Americans (Bolivia, Argentina, Colombia, and Ecuador), 0.7% were Portuguese, 2.4% were Romanian, 1% were Moroccan, 0.7% were Pakistanis, and 2% were of other nationalities.

### Instruments

***The Oviedo Schizotypy Assessment Questionnaire-Revised***.^55^

The Oviedo Schizotypy Assessment Questionnaire-Revised (ESQUIZO-Qr) is a self-report measure developed for the assessment of schizotypal traits in adolescents. It consists of 62 items with a Likert-type response format in five categories (from 1 “totally disagree” to 5 “totally agree”). Its 10 subscales are derived empirically by means of factor analysis, which in turn are grouped into three general dimensions: Reality distortion/Positive schizotypy (eg, Ideas of reference, Magical thinking, Unusual perceptual experiences, and Paranoid ideation), Anhedonia (Physical anhedonia and Social anhedonia), and Social disorganization (Odd thinking and speech, Odd behavior, Lack of close friends, and Excessive social anxiety). Reliability estimates for the subscales ranged from 0.62 to 0.90. In addition, the ESQUIZO-Qr has been administered to representative samples of adolescents and its psychometric properties have been tested in relation to a number of psychometric indicators of mental health (eg, depression, personality disorders, emotional, and behavioral problems).^41,56^

#### Reflective Functioning Questionnaire.

^57,58^ The Reflective Functioning Questionnaire (RFQ) is an 8-item questionnaire that measures two subscales, the certainty (RFQc), and the uncertainty (RFQu) about mental states. The 8 questions are rated from 1 (“totally disagree”) to 7 (“totally agree”). Items were rescored based on the procedure described by the authors of the questionnaire, on a scale from 0 to 3 (for details, see Fonagy et al^57^). High scores on the uncertainty subscale indicate a lack of use of mental states. High scores on the certainty subscale indicate an adaptive level of certainty about mental states. The Spanish translation of the RFQ was used in the present study. Studies focusing on mental health and psychopathology have used the RFQu subscale.^54,59^ In the present study, the reliability estimate of the RFQu total score was 0.78.

#### The Strengths and Difficulties Questionnaire *Self-report Version.*

^60^ The Strengths and Difficulties Questionnaire (SDQ) is a self-report questionnaire that is widely used for the assessment of various emotional and behavioral problems related to mental health in adolescents. The SDQ is comprised of a total of 25 statements divided into five subscales: Emotional symptoms, Conduct problems, Hyperactivity, Peer problems, and Prosocial behavior. The first four subscales result in a Total difficulties score. In this study, we used a Likert-type response format with three options (0 = “Not true,” 1 = “Somewhat true,” 2 = “Certainly true”). The validated Spanish version of the SDQ was used in the present study.^61^ The Spanish version of the SDQ is available on the official website of the SDQ (https://www.sdqinfo.org/py/sdqinfo/b0.py).

#### The Oviedo Infrequency Scale.

^62^ The Oviedo Infrequency Scale (INF-OV) was administered to participants with the goal of identifying those who responded in a random, pseudorandom, or dishonest manner. The INF-OV is a self-report questionnaire composed of 12 items (eg, “The distance between Madrid and Barcelona is greater than the distance between Madrid and New York”) in a 5-point Likert-scale format (from 1 “completely disagree” to 5 “completely agree”). Students with more than three incorrect responses on the INF-OV scale were excluded from the sample. The INF-OV has been used in previous studies.^63–65^

### Procedure

The study was approved by the Ethical Committee for Clinical Research of La Rioja (CEImLAR, PI 552). The survey was conducted in groups of 10–30 students, during school hours, in a specially equipped classroom, using personal computers. The administration was carried out under the supervision of researchers who had been trained in a standard protocol. There were no incentives for participation. Written parental consent was required for students. Participants were informed of the confidentiality of their responses as well as the voluntary nature of the study.

### Data Analyses

First, we calculated descriptive statistics for the measures. Second, considering all variables as continuous, Pearson correlations were calculated between the ESQUIZO-Qr dimensions, the SDQ subscales, and the RFQ uncertainty total score. The prosocial behavior subscale of the SDQ, which assesses strengths, was not used in the present study. Third, two DAGs were computed: (a) within the schizotypal subscales; and (b) between the schizotypal higher-order dimension, RFQ uncertainty, and SDQ subscales. A DAG is a mathematical object that provides the graphical representation of a Bayesian network. DAGs are graphical structures underlying Bayesian networks that model the overall dependence structure of multiple variables. The set of variables of interest is represented as nodes (eg, variables) connected by directed edges. A DAG is directed (from cause to effect) and acyclic (there is no path that starts from a node and returns to the same node, always following edges in the direction of the arrow).^66^ An analysis based on DAGs has the advantage over one based on Markov random fields, in that it suggests explicit directions for the causal relationships among the variables used in the study. Given the assumptions and limitations of DAGs, upstream nodes are potential causes of downstream nodes. However, the direction of the arrows provides no information about the sign of the association, eg, we cannot take the simple network A -> B to necessarily imply that the presence of A increases the probability that B is also present, rather that it changes the probability of B being present, and could also decrease it. For the estimation of the DAG, in the present article, we used the approach and the scripts published by McNally et al,^35^ using the hill-climbing algorithm from the R package *bnlearn*.^67^ The hill-climbing algorithm uses a random procedure to go through models and evaluate their fit to the data until it finds one that fits best (locally). The procedure is not guaranteed to find a global optimum.

In order to draw more robust conclusions, we assessed the stability of the psychological networks. We bootstrapped 1000 samples and computed a network for each of them, and a consensus network of all 1000 networks to obtain the final, resultant estimated network. First, the strength of an edge does not typically refer to how often it appears in a bootstrapped (or even sampled) collection of DAGs, but rather to the magnitude of the coefficient in a structural equation model describing the probabilistic relationship of a node to its parents (ie, how strong the effect of one variable is on another). If an edge appeared in at least 85% of these networks, we retained it in the final consensus network. Second, we determined the probability direction of each edge in the 1000 bootstrapped networks. If an edge connecting two nodes was estimated in at least 51% of the fitted networks, this direction then appeared in the final network. Thus, all estimated edges in the final networks appeared in at least 85% of the fitted networks and were pointing in the given direction in a least 51% of the fitted networks. Further information on the analysis can be found elsewhere.^35,66^ R scripts for the Bayesian network analysis of the study are shown in the Supplementary material. Datasets can be found at the following link: https://osf.io/h4eu7/. SPSS 28 and R 4.3.2 version were used for statistical analyses.

## Results

### Descriptive Statistics

Table 1 shows the descriptive statistics of the subscales and total scores for the entire sample. Table 2 depicts the Pearson correlations of the variables used in the present study. Schizotypal facets, especially the Positive and Social disorganization dimensions, were moderately associated with psychopathology and RF impairments.

**Table 1.** Descriptive Statistics of the Measures

	Mean	SD	Skewness	Kurtosis	Min.	Max.
ESQ-Q Ideas of reference	6.74	2.89	1.14	1.03	4.00	20.00
ESQ-Q Magical thinking	8.28	3.28	1.20	1.44	5.00	25.00
ESQ-Q Unusual perceptual experiences	10.95	4.90	1.67	2.79	7.00	35.00
ESQ-Q Odd thinking and language	15.24	5.04	0.24	−0.45	6.00	30.00
ESQ-Q Paranoid ideation	8.33	3.50	1.27	1.65	5.00	25.00
ESQ-Q Physical anhedonia	16.35	4.05	0.24	−0.30	8.00	30.00
ESQ-Q Social anhedonia	17.23	4.55	0.91	1.05	10.00	37.00
ESQ-Q Odd behavior	7.59	2.98	1.09	1.18	4.00	20.00
ESQ-Q No close friends	10.11	3.90	0.30	−0.58	4.00	20.00
ESQ-Q Excessive social anxiety	17.25	5.70	0.46	−0.05	7.00	35.00
SDQ Emotional problems	3.55	2.45	0.49	−0.53	0.00	10.00
SDQ Conduct problems	1.97	1.65	0.91	0.81	0.00	9.00
SDQ Peer problems	1.51	1.56	1.36	2.07	0.00	9.00
SDQ Hiperactivity	4.31	2.17	0.10	−0.52	0.00	10.00
RFQ Uncertainty	3.89	3.57	1.25	1.49	0.00	18.00

*Note:* ESQ-Q, Oviedo Schizotypy Assessment Questionnaire-Revised; RFQ, Reflective Functioning Questionnaire; SDQ, Strengths and Difficulties Questionnaire.

**Table 2.** Pearson’s Correlations Between Schizotypal Traits, Psychopathology, and Hypomentalizing

	1	2	3	4	5	6	7	8	9	10	11	12	13	14	15
ESQ-Q Ideas of reference (1)	1														
ESQ-Q Magical thinking (2)	0.507**	1													
ESQ-Q Unusual perceptual experiences (3)	0.561**	0.594**	1												
ESQ-Q Odd thinking and language (4)	0.342**	0.375**	0.450*	1											
ESQ-Q Paranoid ideation (5)	0.433**	0.457**	0.513**	0.415**	1										
ESQ-Q Physical anhedonia (6)	−0.048	−0.04	−0.019	−0.004	0.054*	1									
ESQ-Q Social anhedonia (7)	.225**	.186**	.296**	.301**	.388**	.203**	1								
ESQ-Q Odd behavior (8)	0.404**	0.357**	0.485**	0.409**	0.517**	−0.052*	0.462**	1							
ESQ-Q No close friends (9)	0.253**	0.266**	0.301**	0.387**	0.421**	−0.041	0.427**	0.429**	1						
ESQ-Q Excessive social anxiety (10)	0.203**	0.182**	0.267**	0.415**	0.282**	−0.024	0.377**	0.368**	0.343**	1					
SDQ Emotional problems (11)	0.222**	0.268**	0.337**	0.448**	0.344**	−0.068**	0.266**	0.333**	0.402**	0.510**	1				
SDQ Conduct problems (12)	0.243**	0.238**	0.320**	0.319**	0.404**	0.084**	0.209**	0.259**	0.250**	0.079**	0.182**	1			
SDQ Peer problems (13)	0.246**	0.199**	0.325**	0.273**	0.474**	−0.015	0.481**	0.502**	0.413**	0.336**	0.350**	0.244**	1		
SDQ Hiperactivity (14)	0.154**	0.198**	0.247**	0.550**	0.223**	0.02	0.091**	0.190**	0.177**	0.154**	0.217**	0.368**	0.122**	1	
RFQ Uncertainty (15)	0.261**	0.248**	0.364**	0.432**	0.339**	−0.05	0.170**	0.285**	0.332**	0.259**	0.416**	0.369**	0.239**	0.320**	1

*Note:* ESQ-Q, Oviedo Schizotypy Assessment Questionnaire-Revised; SDQ: Strengths and Difficulties Questionnaire; RFQ: Reflective Functioning Questionnaire.

*P < .05,

**P < .01.

### DAG of Schizotypal Traits

**Fig. 1.**
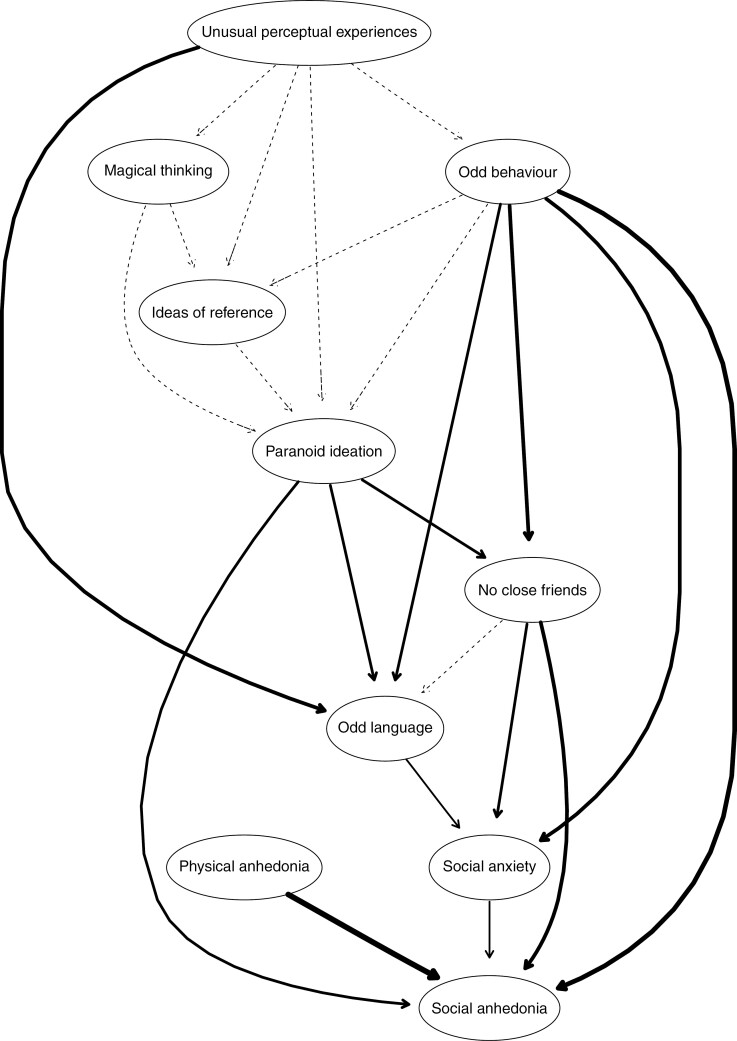
The directed acyclic graph (DAG) of schizotypal traits measured by the Oviedo Schizotypy Assessment Questionnaire-Revised. This graphical representation shows the consensus network of 1000 bootstrapped models. All estimated edges in the estimated network appeared in at least 85% of the fitted networks and were pointing in the given direction in a least 51% of the fitted networks. Thick arrows indicate high directional probabilities, and thin arrows indicate low directional probabilities.

Figure 1 presents the estimated DAG for schizotypal traits. We can observe that Unusual perceptual experiences are placed at the top of the network as potential causes of downstream nodes related to the Social disorganization and Anhedonia facets. Anhedonia is placed at the bottom of the estimated network. The DAG also suggests that the Positive schizotypal nodes (ie, Unusual perceptual experiences, Magical thinking, Ideas of reference, and Paranoid ideation) have a putative causal relationship with Odd thinking and language and Anhedonia facets. Paranoid ideation and Odd behavior are directly related to Social anhedonia and also indirectly via Odd thinking and language and No close friends nodes. Thus, putative causal relationships are presented between Positive schizotypy and Social disorganization dimensions.

### DAG of Schizotypal Dimensions, Psychopathology, and Hypomentalizing

**Fig. 2.**
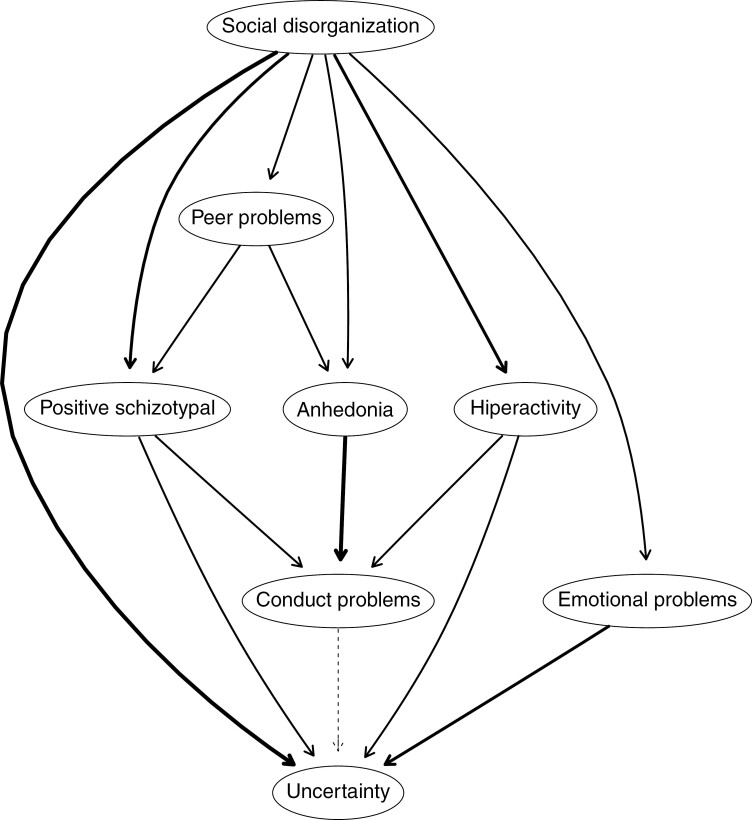
The directed acyclic graph (DAG) of schizotypal higher-order dimensions, psychopathology, and hypomentalizing. Positive schizotypy (Ideas of reference, Magical thinking, Unusual perceptual experiences, and Paranoid ideation), Anhedonia (Physical anhedonia and Social anhedonia), and Social disorganization (Odd thinking and speech, Odd behavior, Lack of close Friends, and Excessive social anxiety). This graphical representation shows the consensus network of 1000 bootstrapped models. All estimated edges in the estimated network appeared in at least 85% of the fitted networks and were pointing in the given direction in a least 51% of the fitted networks. Thick arrows indicate high directional probabilities, and thin arrows indicate low directional probabilities.

Figure 2 presents the estimated DAG for schizotypal higher-order dimensions, psychopathology, and hypomentalizing. Several aspects should be noted: (a) the Social disorganization dimension is placed at the top of the network; (b) Hypomentalizing is placed at the bottom of the network; (c) the Social disorganization dimension shows a putative causal relationship with Positive schizotypy, psychopathology, and RF impairments; (d) Social disorganization shows a putative causal relationship with emotional and behavioral problems; (e) Social disorganization shows a putative causal relationship with RF impairments and also indirectly via other nodes; (f) Reality distortion dimension shows a putative causal relationship with externalizing psychopathology and RF impairments.

In addition, in order to test the robustness of the findings and to support the reliability of the results, the DAG of schizotypal higher-order dimensions, psychopathology, and certainty was estimated (see Supplementary figure S1).

## Discussion

Despite the large body of research on schizotypal traits, critical gaps, and caveats remain unsolved. For instance, few studies have employed the Bayesian network to gain insight into the possible directions of the interdependencies among the variables related to broadly defined mental risk states during adolescence. Thus, the present study examined the putative causal relationship within schizotypal facets, as well as between schizotypal dimensions, psychopathology, and reflective functioning (RF) impairments, in a representative sample of adolescents from the general population. This study is the first to use Bayesian networks to examine directional interdependencies among schizotypal facets, psychopathology, and mentalizing impairments. This study may allow us not only to better understand schizotypal traits and its comorbidities with psychopathology and hypomentalizing, but also to explore tentative causal pathways to develop or refine prevention strategies for youth at increased risk for SSPDs outcomes.

First, as shown by the bivariate correlations, schizotypal traits were moderately related to internalizing and externalizing dimensions of psychopathology and to RF impairments during adolescence. On the one hand, Emotional problems were associated with Odd thinking and language and Positive schizotypy, whereas Social disorganization facets were closely related to Peer and Conduct problems. Previous studies have shown that schizotypal traits and psychotic-like experiences are associated with emotional dysregulation and behavioral problems.^42,68–70^ On the other hand, Unusual perceptual experiences, Odd thinking and language, and Paranoid ideation showed the strongest relationship with hypomentalizing. Prior research conducted in adolescents has shown that schizotypal traits are related to RF difficulties.^54,71^ For instance, Interpersonal schizotypal manifestations pertaining to Social anxiety are associated with high mentalizing uncertainty.^54^ These results indicate that there is a moderate relationship between schizotypal traits, mental health problems, and RF impairments at a subclinical level. These mental health difficulties and RF impairments appear to be characteristic along the extended psychosis phenotype.^40,52,72^

Second, we have observed that the Positive schizotypal traits (eg, Magical thinking, Unusual perceptual experiences, and Paranoid ideation) were placed at the top of the estimated network, suggesting that these facets show putative causal relationships with the Social disorganization and Negative schizotypal facets. The directions of the schizotypal traits give us clues as to which trait associations may be clinically significant. Thus, according to Lenzenweger,^73^ it is important to determine whether one of the schizotypal facets has a genuine causal relationship with another. Prior evidence suggests that critical interactions between schizotypal traits occur during adolescence and predict the unfolding of clinically significant psychotic symptoms.^74–76^ For instance, Debbané et al,^75^ in a longitudinal study, found that the relationship between disorganization features and positive schizotypy may play a central role in establishing risk for psychosis during adolescence. Other studies found that psychosis-like experiences were related to the development of secondary beliefs and appraisals.^77^ These results are in line with the model proposed by Paul E. Meehl^78^ who suggested that slippage (both cognitive and affective), rather than anhedonia, constitutes a key component of schizophrenia. These observations highlight a potentially critical relationship between Positive and Social disorganization schizotypal dimensions and an increased risk for psychosis during adolescence.

Third, probabilistic DAG suggested that the schizotypal dimensions were related to psychopathology and mentalizing impairments. As previously mentioned, DAG allows us to capture the dependence structure of multiple variables and, when used appropriately, to draw inferences based on putative causal relationships.^31^ In relation to schizotypal dimensions, previous work, using other approaches, has found similar results. For instance, in a 10-year follow-up study, Dominguez et al^74^ found that early expression of negative/disorganized symptoms predicted psychotic experiences and subsequent clinical psychosis. The results also implied that the disorganized features were associated with psychotic disorder at the population level. In relation to schizotypal facets and RF, and according to Debbané and Barrantes-Vidal^79^ cognitive and interpersonal aberrations in the context of schizotypal traits, may disrupt the normative development of RF processes during adolescence and impair the ability to understand mental states. Mentalizing is a capacity that is acquired in social relationships, and its function is to facilitate social navigation through an understanding of mental states that govern behavior.^80^ Interestingly, the Bayesian network lends evidence to the idea that mentalizing is a uniquely dependent regular, regulated, and reciprocal social interaction in order to appropriately develop itself.^81^ This result is also in line with the idea that some facets of schizotypy may alter the development of mentalizing, which in turn augments the risk of the emergence of dysfunctional mental state attributions and the genesis of delusional belief systems.^54,82^ Some recent clinical work suggests that intervening in mentalizing may serve as a protective factor for the functional impairments associated with risk for psychosis.^48,49^ Critically, however, these developmental hypotheses need to be tested in longitudinal samples, and the first reports using such samples would appear to motivate further inquiry in relating disorganization through RF towards increased risk for psychosis. These findings allow for the possibility that different putative causal relationships may operate through different entry points in the network (eg, different stages of development), and this information may have useful implications for prevention and psychological treatments in both educational and clinical settings.^83^

Fourth, DAG using schizotypal traits (figure 1), found that Positive schizotypal traits are all above Social disorganization facets, while in the DAG (figure 2), Social disorganization is above Positive schizotypal traits. These results may have several tentative explanations. First, Bayesian networks are probabilistic graphical models widely employed to understand dependencies in high-dimensional data,^29^ with strong assumptions and clear limitations and strengths. Second, in the first Bayesian network we use schizotypal facets at the subscale level, while in the second we use second-order dimensions of schizotypal personality. So, the level of analysis changes across DAGs. Third, in the second Bayesian network, psychopathology, and mentalization variables are introduced, an aspect that may affect the reconfiguration of the estimated network found. For example, mentalization might be closely related to Social disorganization. Hypothetically, if other facets had been introduced into the estimated network a different configuration would have been found. In this study, schizotypal traits have been characterized as a complex dynamic system in which symptoms (behaviors, affects, mental states, etc.), are directly connected to one another in a network structure and depend on the number and type of nodes introduced into the network.

Our study had some limitations. Due to the cross-sectional design used and the high potential for biases, particularly uncontrolled confounding variables (eg, causal sufficiency), causal conclusions cannot be drawn from this study. Secondly, the information was based on self-reported questionnaires only. The variables examined here depend on the instruments used (eg, Anhedonia is measured by two subscales of the ESQUIZO-Qr), which limits the generalizability of our findings. In addition, measures used in the present study were created based on a latent variable framework.^84^ Thirdly, learning DAGs from data and their causal interpretation rely on strong assumptions, such as causal sufficiency (no unmeasured confounders), faithfulness, and the causal Markov property.^30^ Thus, drawing conclusions from the data may require caution. For instance, the hill-climbing-based procedure is not guaranteed to return DAGs from the same equivalence class with equal probability, potentially leading to biased results. These limitations, together with the heuristic nature of the procedure used, do not necessarily capture the distribution of the edges in the network. DAG models are powerful tools for improving communication and guiding research.

## Conclusions

This study is the first to comprehensively examine the Bayesian network structure within schizotypal traits as well as the relationships among schizotypal dimensions, psychopathology, and hypomentalizing during adolescence. This study also provides a deeper understanding of the schizotypal personality and its links to psychopathology and dysfunction in RF. Understanding the network structures of schizotypal traits, as an emergent property that arises from causal relations among facets (behaviors, cognitions, and emotions),^85^ may help to prevent SSPDs and mental health disorders. Finally, studying the interrelationship between schizotypal traits, mental health problems or mentalizing impairments, prior to clinical disorders, may elucidate the mechanisms for the development of psychosis. Gaining a better understanding of schizotypes by adding new psychometric models opens the door to exploring the potential mechanisms involved in the etiology of psychosis, and thus, in its clinical approach.

## Supplementary Material

Supplementary material is available at https://academic.oup.com/schizophreniabulletin/.

sbae041_suppl_Supplementary_Figures_S1

## References

[1] O’Hare K, Watkeys O, Whitten T, et al Cumulative environmental risk in early life: associations with schizotypy in childhood. Schizophr Bull. 2023;49(2):244–254. doi: https://doi.org/10.1093/SCHBUL/SBAC160 36302227 PMC10016419

[2] Green MJ, O’Hare K, Laurens KR, et al Developmental profiles of schizotypy in the general population: a record linkage study of Australian children aged 11–12 years. Br J Clin Psychol. 2022;61(3):836–858. doi: https://doi.org/10.1111/BJC.12363 35229307 PMC9541481

[3] Van Os J, Pries LK, Ten Have M, et al Context v. algorithm: evidence that a transdiagnostic framework of contextual clinical characterization is of more clinical value than categorical diagnosis. Psychol Med. 2023;53(5):1825–1833. doi: https://doi.org/10.1017/S0033291721003445 37310330 PMC10106290

[4] Debbané M, Fonseca Pedrero E. Early schizotypy and risk: the need for integrating developmental dynamics. Schizophr Bull. 2023;49(2):234–236. doi: https://doi.org/10.1093/SCHBUL/SBAC161 36259928 PMC10016389

[5] Meehl PE. Schizotaxia, schizotypy, schizophrenia. Am Psychol. 1962;17(12):827–838. doi: https://doi.org/10.1037/h0041029

[6] Lenzenweger MF. Thinking clearly about schizotypy: hewing to the schizophrenia liability core, considering interesting tangents, and avoiding conceptual quicksand. Schizophr Bull. 2015;41(Suppl 2):S483–S491. 25810061 10.1093/schbul/sbu184 PMC4373631

[7] Fonseca-Pedrero E, Debbané M, Rodríguez-Testal JF, Cohen AS, Docherty AR, Ortuño-Sierra J. Schizotypy: the way ahead. Psicothema. 2021;33(1):16–27. doi: https://doi.org/10.7334/psicothema2019.285 33453732

[8] Fonseca-Pedrero E, Debbané M, Ortuño-Sierra J, et al The structure of schizotypal personality traits: a cross-national study. Psychol Med. 2018;48:451–462. doi: https://doi.org/10.1017/S0033291717001829 28712364

[9] Debbané M, Eliez S, Badoud D, Conus P, Flückiger R, Schultze-Lutter F. Developing psychosis and its risk states through the lens of schizotypy. Schizophr Bull. 2015;41:S396–S407. 25548386 10.1093/schbul/sbu176 PMC4373628

[10] Barrantes-Vidal N, Grant P, Kwapil T. The role of schizotypy in the study of the etiology of schizophrenia spectrum disorders. Schizophr Bull. 2015;41:S408–S416. 25810055 10.1093/schbul/sbu191 PMC4373635

[11] Linscott RJ, van Os J. An updated and conservative systematic review and meta-analysis of epidemiological evidence on psychotic experiences in children and adults: on the pathway from proneness to persistence to dimensional expression across mental disorders. Psychol Med. 2013;43:1133–1149. 22850401 10.1017/S0033291712001626

[12] Van Os J, Reininghaus U. Psychosis as a transdiagnostic and extended phenotype in the general population. World Psychiatry. 2016;15(2):118–124. doi: https://doi.org/10.1002/wps.20310 27265696 PMC4911787

[13] Lenzenweger MF. Schizotypy 17 years on: psychotic symptoms in midlife. J Abnorm Psychol. 2021;130(4):399–412. doi: https://doi.org/10.1037/ABN0000680 34180704

[14] Flückiger R, Michel C, Hubl D, et al Psychosis-predictive value of self-reported schizotypy in a clinical high-risk sample. J Abnorm Psychol. 2016;125(7):923–932. doi: https://doi.org/10.1037/abn0000192 27583768

[15] Radua J, Ramella-Cravaro V, Ioannidis JPA, et al What causes psychosis? An umbrella review of risk and protective factors. World Psychiatry. 2018;17(1):49–66. doi: https://doi.org/10.1002/wps.20490 29352556 PMC5775150

[16] Fusar-Poli P, Cappucciati M, Rutigliano G, et al At risk or not at risk? A meta-analysis of the prognostic accuracy of psychometric interviews for psychosis prediction. World Psychiatry. 2015;14(3):322–332. doi: https://doi.org/10.1002/wps.20250 26407788 PMC4592655

[17] Fisher HL, Caspi A, Poulton R, et al Specificity of childhood psychotic symptoms for predicting schizophrenia by 38 years of age: a birth cohort study. Psychol Med. 2013;43:2077–2086. 23302254 10.1017/S0033291712003091 PMC3758773

[18] Borsboom D. A network theory of mental disorders. World Psychiatry. 2017;16:5–13. doi: https://doi.org/10.1002/wps.20375 28127906 PMC5269502

[19] Borsboom D, Cramer AOJ. Network analysis: an integrative approach to the structure of psychopathology. Annu Rev Clin Psychol. 2013;9:91–121. doi: https://doi.org/10.1146/annurev-clinpsy-050212-185608 23537483

[20] Fonseca-Pedrero E. Network analysis in psychology. Papeles del Psicol. 2018;39(1):1–12. doi: https://doi.org/10.23923/pap.psicol2018.2852

[21] Li G, Wang L, Cao C, et al An exploration of the DSM-5 posttraumatic stress disorder symptom latent variable network. Eur J Psychotraumatol. 2020;11(1). doi: https://doi.org/10.1080/20008198.2020.1759279 PMC7448915 32922682

[22] Borsboom D, Deserno MK, Rhemtulla M, et al Network analysis of multivariate data in psychological science. Nat Rev Methods Prim. 2021;1(1):1–18. doi: https://doi.org/10.1038/s43586-021-00055-w

[23] Bringmann LF, Albers C, Bockting C, et al Psychopathological networks: theory, methods and practice. Behav Res Ther. 2022;149:104011. doi: https://doi.org/10.1016/J.BRAT.2021.104011 34998034

[24] Isvoranu A-M, Borsboom D, van Os J, Guloksuz S. A network approach to environmental impact in psychotic disorder: brief theoretical framework. Schizophr Bull. 2016;42(4):870–873. doi: https://doi.org/10.1093/schbul/sbw049 27179124 PMC4903069

[25] Kuranova A, Wigman JTW, Menne-Lothmann C, et al Network dynamics of momentary affect states and future course of psychopathology in adolescents. PLoS One. 2021;16(3):e0247458. doi: https://doi.org/10.1371/JOURNAL.PONE.0247458 33661971 PMC7932519

[26] Wüsten C, Schlier B, Jaya ES, et al; Genetic Risk and Outcome of Psychosis (GROUP) Investigators. Psychotic experiences and related distress: a cross-national comparison and network analysis based on 7141 participants from 13 countries. Schizophr Bull. 2018;44(6):1185–1194. doi: https://doi.org/10.1093/schbul/sby087 29982814 PMC6192474

[27] McNally RJ. Can network analysis transform psychopathology? Behav Res Ther. 2016;86:95–104. doi: https://doi.org/10.1016/j.brat.2016.06.006 27424882

[28] Epskamp S, Fried E. A tutorial on regularized partial correlation networks. Psychol Methods. 2018;23:617–634. doi: https://doi.org/10.1037/met0000167 29595293

[29] Kuipers J, Suter P, Moffa G. Efficient sampling and structure learning of Bayesian networks. J Comput Graph Stat. 2022;31(3):639–650. doi: https://doi.org/10.1080/10618600.2021.2020127

[30] Dawid AP. Beware of the DAG! *Proceedings of Workshop on Causality: Objectives and Assessment at NIPS* 2008. 2010;6:59–86. http://proceedings.mlr.press/v6/dawid10a/dawid10a.pdf

[31] Moffa G, Catone G, Kuipers J, et al Using directed acyclic graphs in epidemiological research in psychosis: an analysis of the role of bullying in psychosis. Schizophr Bull. 2017;43(6):1273–1279. doi: https://doi.org/10.1093/SCHBUL/SBX013 28521005 PMC5737513

[32] Moffa G, Kuipers J, Carrà G, et al Longitudinal symptomatic interactions in long-standing schizophrenia: a novel five-point analysis based on directed acyclic graphs. Psychol Med. 2023;53(4):1371–1378. doi: https://doi.org/10.1017/S0033291721002920 34348816 PMC10009394

[33] Kuipers J, Moffa G, Kuipers E, Freeman D, Bebbington P. Links between psychotic and neurotic symptoms in the general population: an analysis of longitudinal British National Survey data using Directed Acyclic Graphs. Psychol Med. 2019;49(3):388–395. doi: https://doi.org/10.1017/S0033291718000879 29808787

[34] Abplanalp SJ, Lee J, Horan WP, Kern RS, Penn DL, Green MF. A Bayesian network approach to social and nonsocial cognition in schizophrenia: are some domains more fundamental than others? Schizophr Bull. 2023;49:997–1006. doi: https://doi.org/10.1093/schbul/sbad012 36869810 PMC10318874

[35] McNally RJ, Mair P, Mugno BL, Riemann BC. Comorbid obsessive-compulsive disorder and depression: a Bayesian network approach. Psychol Med. 2017;47:1204–1214. doi: https://doi.org/10.1017/S0033291716003287 28052778

[36] Morosan L, Fonseca-Pedrero E, Debbané M. Network analysis of reflective functioning and conduct problems during adolescence. Psychol Violence. 2020;10:300–311. doi: https://doi.org/10.1037/vio0000258

[37] Pearl J. Causality: Models, Reasoning, and Inference. 2nd ed. Cambridge, UK: Cambridge University Press; 2009.

[38] Foraita R, Spallek J, Zeeb H. Directed acyclic graphs. In: Ahrens W, Pigeot I, eds. Handbook of Epidemiology. 2nd ed. New York: Springer; 2014:1481–1517. doi: https://doi.org/10.1007/978-0-387-09834-0_65

[39] Fusar-Poli P, Nelson B, Valmaggia L, Yung AR, McGuire PK. Comorbid depressive and anxiety disorders in 509 individuals with an at-risk mental state: impact on psychopathology and transition to psychosis. Schizophr Bull. 2014;40:120–131. 23180756 10.1093/schbul/sbs136 PMC3885287

[40] Häfner H, Maurer K, Löffler W, An der Heiden W, Hambrecht M, Schultze-Lutter F. Modeling the early course of schizophrenia. Schizophr Bull. 2003;29(2):325–340. doi: https://doi.org/10.1093/OXFORDJOURNALS.SCHBUL.A007008 14552507

[41] Fonseca-Pedero E, Ortuño-Sierra J, Inchausti F, Rodríguez-Testal JF, Debbané M. Beyond clinical high-risk state for psychosis: the network structure of multidimensional psychosis liability in adolescents. Front Psychiatry. 2020;10:967. doi: https://doi.org/10.3389/fpsyt.2019.00967 32116811 PMC7026502

[42] Armando M, Nelson B, Yung AR, et al Psychotic-like experiences and correlation with distress and depressive symptoms in a community sample of adolescents and young adults. Schizophr Res. 2010;119(1–3):258–265. doi: https://doi.org/10.1016/j.schres.2010.03.001 20347272

[43] Kelleher I, Keeley H, Corcoran P, et al Clinicopathological significance of psychotic experiences in non-psychotic young people: evidence from four population-based studies. Br J Psychiatry. 2012;201:26–32. doi: https://doi.org/10.1192/bjp.bp.111.101543 22500011

[44] Staines L, Healy C, Coughlan H, et al Psychotic experiences in the general population, a review; definition, risk factors, outcomes and interventions. Psychol Med. 2022;52(15):3297–3308. doi: https://doi.org/10.1017/S0033291722002550 PMC9772919 36004805

[45] Solmi M, Soardo L, Kaur S, et al Meta-analytic prevalence of comorbid mental disorders in individuals at clinical high risk of psychosis: the case for transdiagnostic assessment. Mol Psychiatry. 2023;28:2291–2300. doi: https://doi.org/10.1038/S41380-023-02029-8 37296309 PMC10611568

[46] Yung AR, Lin A. Psychotic experiences and their significance. World Psychiatry. 2016;15(2):130–131. doi: https://doi.org/10.1002/WPS.20328 27265701 PMC4911755

[47] Gin K, Stewart C, Jolley S. A systematic literature review of childhood externalizing psychopathology and later psychotic symptoms. Clin Psychol Psychother. 2021;28(1):56–78. doi: https://doi.org/10.1002/CPP.2493 32681551

[48] Debbané M, Salaminios G, Luyten P, et al Attachment, neurobiology, and mentalizing along the psychosis continuum. Front Hum Neurosci. 2016;10:406. doi: https://doi.org/10.3389/fnhum.2016.00406 27597820 PMC4992687

[49] Armando M, Hutsebaut J, Debbané M. A mentalization-informed staging approach to clinical high risk for psychosis. Front Psychiatry. 2019;10:385. doi: https://doi.org/10.3389/FPSYT.2019.00385 31214062 PMC6555089

[50] Bateman A, Fonagy P. Mentalization-based treatment. Psychoanal Inq. 2013;33(6):595–613. doi: https://doi.org/10.1080/07351690.2013.835170 26157198 PMC4467231

[51] Fonagy P, Gergely G, Jurist E, Target M. Affect Regulation Mentalization and the Development of the Self. London: Other press; 2002.

[52] Debbané M, Salaminios G, Weijers J, Fonagy P, Fonseca-Pedrero E, Armando M. Clinical evaluation and intervention of emerging psychosis: a mentalization-informed perspective. In: Rocca P, Bellino S, eds. Psychosis and Personality Disorders. Cham, Switzerland: Springer; 2022:125–143.

[53] Bartels-Velthuis A, Blijd-Hoogewys EMA, van Os J. Better theory-of-mind skills in children hearing voices mitigate the risk of secondary delusion formation. Acta Psychiatr Scand. 2011;124(3):193–197. doi: https://doi.org/10.1111/J.1600-0447.2011.01699.X 21426312

[54] Salaminios G, Morosan L, Toffel E, et al Associations between schizotypal personality features, mentalizing difficulties and thought problems in a sample of community adolescents. Early Interv Psychiatry. 2021;15(3):705–715. doi: https://doi.org/10.1111/EIP.13011 32573985

[55] Fonseca-Pedrero E, Muñiz J, Lemos-Giráldez S, Paino M, Villazón-García U. ESQUIZO-Q: Cuestionario Oviedo Para La Evaluación de La Esquizotipia [ESQUIZO-Q: Oviedo Questionnaire for Schizotypy Assessment]. Madrid, Spain: TEA ediciones; 2010.

[56] Fonseca-Pedrero E, Ortuño-Sierra J, Muñiz J, Bobes J. Latent profile analysis of psychosis liability in a community-derived sample of adolescents: links with mental health difficulties, suicidal ideation, bipolar-like experiences and psychotic-like experiences. Early Interv Psychiatry. 2018;13:1111–1120. doi:10.1111/eip.12741 30311391

[57] Fonagy P, Luyten P, Moulton-Perkins A, et al Development and validation of a self-report measure of mentalizing: the reflective functioning questionnaire. PLoS One. 2016;11(7):e0158628–e0158678. doi: https://doi.org/10.1371/journal.pone.0158678 27392018 PMC4938585

[58] Badoud D, Luyten P, Fonseca-Pedrero E, Eliez S, Fonagy P, Debbané M. The French version of the reflective functioning questionnaire: validity data for adolescents and adults and its association with non-suicidal self-injury. PLoS One. 2015;10(12):e0145892. doi: https://doi.org/10.1371/journal.pone.0145892 26714319 PMC4694697

[59] Müller S, Wendt LP, Spitzer C, Masuhr O, Back SN, Zimmermann J. A critical evaluation of the Reflective Functioning Questionnaire (RFQ). J Pers Assess. 2022;104(5):613–627. doi: https://doi.org/10.1080/00223891.2021.1981346 34597256

[60] Goodman R. The strengths and difficulties questionnaire: a research note. J Child Psychol Psychiatry. 1997;38:581–586. 9255702 10.1111/j.1469-7610.1997.tb01545.x

[61] Ortuño-Sierra J, Sebastián-Enesco C, Pérez-Albéniz A, Lucas-Molina B, Fonseca-Pedrero E. Spanish normative data of the Strengths and Difficulties Questionnaire in a community-based sample of adolescents: Datos normativos españoles del Cuestionario de capacidades y dificultades (SDQ) en una muestra comunitaria de adolescentes. Int J Clin Health Psychol. 2022;22(3):100328. doi: https://doi.org/10.1016/J.IJCHP.2022.100328 36111263 PMC9442435

[62] Fonseca-Pedrero E, Lemos-Giráldez S, Paino M, Villazón-García U, Muñiz J. Validation of the schizotypal personality questionnaire brief form in adolescents. Schizophr Res. 2009;111:53–60. 19342199 10.1016/j.schres.2009.03.006

[63] Fonseca-Pedrero E, Pérez-Albéniz A, Al-Halabí S, et al PSICE project protocol: evaluation of the unified protocol for transdiagnostic treatment for adolescents with emotional symptoms in school settings. Clínica y Salud. 2023;34:15–22. doi: https://doi.org/10.5093/CLYSA2023A3

[64] Pérez-Albéniz A, Lucas-Molina B, Fonseca-Pedrero E. Parental support and gender moderate the relationship between sexual orientation and suicidal behavior in adolescents. Psicothema. 2023;35(3):248–258. doi: https://doi.org/10.7334/PSICOTHEMA2022.325 37493148

[65] Álvarez-Marín I, Pérez-Albéniz A, Lucas-Molina B, Martínez-Valderrey V, Fonseca-Pedrero E. Development and validation of a brief version of the European Bullying and Cyberbullying Intervention Project Questionnaires (EBIP-Q and ECIP-Q). Psicothema. 2022;34(4):571–581. doi: https://doi.org/10.7334/PSICOTHEMA2022.156 36268962

[66] Jones PJ, Mair P, Riemann BC, Mugno BL, McNally RJ. A network perspective on comorbid depression in adolescents with obsessive-compulsive disorder. J Anxiety Disord. 2018;53:1–8. doi: https://doi.org/10.1016/J.JANXDIS.2017.09.008 29125957

[67] Scutari M. Learning Bayesian Networks with the bnlearn R Package. *J Stat Soft*. 2009;35(3). doi: https://doi.org/10.18637/jss.v035.i03

[68] Fonseca-Pedrero E, Lemos-Giráldez S, Paino M, Muñiz J. Schizotypy, emotional-behavioural problems and personality disorder traits in a non-clinical adolescent population. Psychiatry Res. 2011;190:316–321. 21802744 10.1016/j.psychres.2011.07.007

[69] Kemp KC, Gross GM, Barrantes-Vidal N, Kwapil TR. Association of positive, negative, and disorganized schizotypy dimensions with affective symptoms and experiences. Psychiatry Res. 2018;270:1143–1149. doi: https://doi.org/10.1016/J.PSYCHRES.2018.10.031 30366639

[70] Lewandowski KE, Barrantes-Vidal N, Nelson-Gray RO, Clancy C, Kepley HO, Kwapil TR. Anxiety and depression symptoms in psychometrically identified schizotypy. Schizophr Res. 2006;83(2–3):225–235. doi: https://doi.org/10.1016/J.SCHRES.2005.11.024 16448805

[71] Salaminios G, Sprüngli-Toffel E, Michel C, et al The role of mentalizing in the relationship between schizotypal personality traits and state signs of psychosis risk captured by cognitive and perceptive basic symptoms. Front Psychiatry. 2023;14:1267656. doi: https://doi.org/10.3389/FPSYT.2023.1267656 37810595 PMC10557948

[72] Wigman JTW, de Vos S, Wichers M, van Os J, Bartels-Velthuis AA. A transdiagnostic network approach to psychosis. Schizophr Bull. 2016;43:122–132. doi: https://doi.org/10.1093/schbul/sbw095 27384055 PMC5216855

[73] Lenzenweger MF. Schizotypy, schizotypic psychopathology and schizophrenia. World Psychiatry. 2018;17:25–26. 29352536 10.1002/wps.20479 PMC5775125

[74] Dominguez MD, Saka MC, Lieb R, Wittchen HU, van Os J. Early expression of negative/disorganized symptoms predicting psychotic experiences and subsequent clinical psychosis: a 10-year study. Am J Psychiatry. 2010;167:1075–1082. 20634371 10.1176/appi.ajp.2010.09060883

[75] Debbané M, Badoud D, Balanzin D, Eliez S. Broadly defined risk mental states during adolescence: disorganization mediates positive schizotypal expression. Schizophr Res. 2013;147:153–156. 23570898 10.1016/j.schres.2013.03.012

[76] De Loore E, Gunther N, Drukker M, et al Persistence and outcome of auditory hallucinations in adolescence: a longitudinal general population study of 1800 individuals. Schizophr Res. 2011;127:252–256. 21315559 10.1016/j.schres.2011.01.015

[77] Krabbendam L, Myin-Germeys I, Hanssen M, et al Hallucinatory experiences and onset of psychotic disorder: evidence that the risk is mediated by delusion formation. Acta Psychiatr Scand. 2004;110(4):264–272. 15352927 10.1111/j.1600-0447.2004.00343.x

[78] Meehl PE. Toward an integrated theory of schizotaxia, schizotypy, and schizophrenia. J Personal Disord. 1990;4(1):1–99.

[79] Debbané M, Barrantes-Vidal N. Schizotypy from a developmental perspective. Schizophr Bull. 2015;41(Suppl 2):S386–S395. 25548385 10.1093/schbul/sbu175 PMC4373627

[80] Luyten P, Campbell C, Allison E, Fonagy P. The mentalizing approach to psychopathology: state of the art and future directions. Annu Rev Clin Psychol. 2020;16:297–325. doi: https://doi.org/10.1146/ANNUREV-CLINPSY-071919-015355 32023093

[81] Fonagy P, Bateman AW. Adversity, attachment, and mentalizing. Compr Psychiatry. 2016;64:59–66. doi: https://doi.org/10.1016/J.COMPPSYCH.2015.11.006 26654293

[82] Debbané M, Salaminios G, Luyten P, et al Attachment, neurobiology, and mentalizing along the psychosis continuum. Front Hum Neurosci. 2016;10:22. doi: https://doi.org/10.3389/FNHUM.2016.00406 27597820 PMC4992687

[83] Fonseca-Pedrero E, Díez-Gómez A, Pérez-Albéniz A, Lucas-Molina B, Al-Halabí S, Calvo P. Psychology professionals in educational contexts: an unavoidable necessity. Papeles del Psicólogo/Psychol Papers. 2023;44:112–124. doi: https://doi.org/10.23923/pap.psicol.3018

[84] Hallquist MN, Wright AGC, Molenaar PCM. Problems with centrality measures in psychopathology symptom networks: why network psychometrics cannot escape psychometric theory. Multivariate Behav Res. 2021;56(2):199–223. doi: https://doi.org/10.1080/00273171.2019.1640103 31401872 PMC7012663

[85] Polner B, Faiola E, Urquijo MF, et al The network structure of schizotypy in the general population. Eur Arch Psychiatry Clin Neurosci. 2021;271(4):635–645. doi: https://doi.org/10.1007/s00406-019-01078-x 31646383 PMC8119252

