# Supplementary material for: Schizotypal Traits, Psychopathology, and Reflective Functioning Impairments During Adolescence: A Bayesian Network Approach
Source: Schizophr Bull. 2025 Mar 4;51(Suppl 2):S214–25. doi: 10.1093/schbul/sbae041 (PMC11879529; doi:10.1093/schbul/sbae041)
Supplement: Supplementary file 1 [file sbae041_suppl_supplementary_figures_s1.zip › sbae041_suppl_Supplementary_Figures_S1.odt]

Supplementary Materials

S1. Figure S1. The directed acyclic graph (DAG) of schizotypal higher-order dimensions, psychopathology, and certainty 

S2. R scripts for the network analysis of the study

S1. Supplementary Figure

Figure S1. The directed acyclic graph (DAG) of schizotypal higher-order dimensions, psychopathology, and certainty

Diagrama

Descripción generada automáticamente

The directed acyclic graph (DAG) of schizotypal higher-order dimensions, psychopathology, and certainty 

Note. Positive schizotypy (Ideas of Reference, Magical Thinking, Unusual Perceptual Experiences, and Paranoid Ideation), Anhedonia (Physical Anhedonia and Social Anhedonia), and Social Disorganization (Odd Thinking and Speech, Odd Behaviour, Lack of Close Friends, and Excessive Social Anxiety). 

This graphical representation shows the consensus network of 1,000 bootstrapped models. All estimated edges in the estimated network appeared in at least 85% of the fitted networks and were pointing in the given direction in a least 51% of the fitted networks. Thick arrows indicate high directional probabilities, thin arrows low directional probabilities.

S2. R scripts for the network analysis of the study

library(bnlearn)

library(psychTools)

library(haven)

library(mgm)

library(qgraph)

library(networktools)

library(dplyr)

library(ggplot2)

library(Hmisc)

library(bootnet)

library(qgraph)

library(networktools)

library(mgm)

library(corrplot)

library(Rgraphviz)

##############FIRST DAG: SCHIZOTYPAL TRAITS######################

##-------------------- data import and data manipulation -----------------------

DAG1 <- read_sav("data1.sav")

View(DAG1)

names(DAG1) <- c("Ideas of reference","Magical thinking",

                 "Unusual perceptual experiences","Odd language",

                   "Paranoid ideation","Physical anhedonia",

                 "Social anhedonia","Odd behaviour", 

                   "No close friends", "Social anxiety")

netdata <- as.data.frame(apply(DAG1, 2, as.numeric)) ## convert to numerics

## ---------------------------- Bayesian network schizotypal -------------------

Bayeisannetwork <- hc(netdata, restart = 50, perturb = 100) ## hc gives directed graph

Bayeisannetwork

bnlearn::score(Bayeisannetwork, data = netdata) ## global network score

astr <- arc.strength(Bayeisannetwork, netdata, "bic-g") ## connection strength

astr[order(astr[,3]), ] ## sorted edge strength from strongest to weakest

dev.new()

bnplot<-strength.plot(Bayeisannetwork,astr, shape = "ellipse")

## Learn 1000 network structures (keep the number of restarts and perturbations considerablly low)

set.seed(123)

bootnet <- boot.strength(netdata, R = 1000, algorithm = "hc", 

                         algorithm.args = list(restart = 5, perturb = 10), debug = TRUE)

head(bootnet)

## filter the ones with a strength larger than 0.85 and a direction probability > 0.5

bootnet[bootnet$strength > 0.85 & bootnet$direction > 0.5, ]

## net1: build the average network using a 0.85 threshold

avgnet1 <- averaged.network(bootnet, threshold = 0.85)

avgnet1

bnlearn::score(avgnet1, data = netdata)

astr1 <- arc.strength(avgnet1, netdata, "bic-g") 

dev.new()

bnplot1<-strength.plot(avgnet1, astr1, shape = "ellipse")

## use 0.85 threshold, edge strenghts are determined by direction probability

boottab2 <- bootnet[bootnet$strength > 0.85 & bootnet$direction > 0.5, ] 

boottab2

astr5 <- boottab2   

astr5$strength <- astr5$direction  

bnplot2<-strength.plot(avgnet1, astr5, shape = "ellipse") 

#####SECOND DAG: schizotypal traits, RF AND PSYCHOPAHOLOGY#####

##-------------------- data import and data manipulation -----------------------

DAG2 <- read_sav("data2.sav")

View(DAG2)

names(DAG2) <- c("Positive schizotypal", "Social disorganization", "Uncertainty",

                 "Emotional problems","Conduct problems",

                 "Peer problems","Hiperactivity","Anhedonia")

netdata1 <- as.data.frame(apply(DAG2, 2, as.numeric)) ## convert to numerics

## --------------- Bayesian network schizotypal, RF, SDQ -------------------

Bayeisannetwork1 <- hc(netdata1, restart = 50, perturb = 100) ## hc gives directed graph

Bayeisannetwork1

bnlearn::score(Bayeisannetwork1, data = netdata1) ## global network score

astr1 <- arc.strength(Bayeisannetwork1, netdata1, "bic-g") ## connection strength

astr1[order(astr1[,3]), ] ## sorted edge strength from strongest to weakest

dev.new()

bnplot1<-strength.plot(Bayeisannetwork1,astr1, shape = "ellipse")

## Learn 1000 network structures (keep the number of restarts and perturbations considerablly low)

set.seed(123)

bootnet1 <- boot.strength(netdata1, R = 1000, algorithm = "hc", 

                         algorithm.args = list(restart = 5, perturb = 10), debug = TRUE)

head(bootnet1)

## filter the ones with a strength larger than 0.85 and a direction probability > 0.5

bootnet1[bootnet1$strength > 0.85 & bootnet1$direction > 0.5, ]

## net1: build the average network using a 0.85 threshold

avgnet2 <- averaged.network(bootnet1, threshold = 0.85)

avgnet2

bnlearn::score(avgnet2, data = netdata1)

astr1 <- arc.strength(avgnet2, netdata1, "bic-g")

dev.new()

bnplot2<-strength.plot(avgnet2, astr1, shape = "ellipse")

## use 0.85 threshold, edge strenghts are determined by direction probability

boottab3 <- bootnet1[bootnet1$strength > 0.85 & bootnet1$direction > 0.5, ] 

boottab3

astr6 <- boottab3   

astr6$strength <- astr6$direction  

bnplot3<-strength.plot(avgnet2, astr6, shape = "ellipse")
